# Analysis of Polygonal Computer Model Parameters and Influence on Fabric Drape Simulation

**DOI:** 10.3390/ma14216259

**Published:** 2021-10-21

**Authors:** Slavenka Petrak, Maja Mahnić Naglić, Dubravko Rogale, Jelka Geršak

**Affiliations:** 1 Department of Clothing Technology, Faculty of Textile Technology, University of Zagreb, 10 000 Zagreb, Croatia; maja.mahnic@ttf.unizg.hr (M.M.N.); dubravko.rogale@ttf.unizg.hr (D.R.); 2 Research and Innovation Centre for Design and Clothing Science, Faculty of Mechanical Engineering, University of Maribor, 2000 Maribor, Slovenia; jelka.gersak@uni-mb.si

**Keywords:** fabric, drape, physical and mechanical properties, polygonal model, 3D simulation

## Abstract

Contemporary CAD systems enable 3D clothing simulation for the purpose of predicting the appearance and behavior of conventional and intelligent clothing in real conditions. The physical and mechanical properties of the fabric and the simulation parameters play an important role in this issue. The paper presents an analysis of the parameters of the polygonal computer model that affect fabric drape simulation. Experimental research on physical and mechanical properties were performed for nine fabrics. For this purpose, the values of the parameters for the tensile, bending, shear, and compression properties were determined at low loads, while the complex deformations were analyzed using Cusick drape meter devices. The fabric drape simulations were performed using the 2D/3D CAD system for a computer clothing design on a disk model, corresponding to real testing on the drape tester in order to allow a correlation analysis between the values of drape parameters of the simulated fabrics and the realistically measured values for each fabric. Each fabric was simulated as a polygonal model with a variable related to the side length of the polygon to analyze the influence of the polygon size, i.e., mesh density, on the model behavior in the simulation. Based on the simulated fabric drape shape, the values of the areas within the curves necessary to calculate the drape coefficients of the simulated fabrics were determined in the program for 3D modelling. The results were statistically processed and correlations between the values of the drape coefficients and the optimal parameters for simulating certain physical and mechanical properties of the fabric were determined. The results showed that the mesh density of the polygonal model is an important parameter for the simulation results.

## 1. Introduction

The behavior of textile materials from the aspect of mechanical properties, such as tensile, bending, shear, and compression properties, can be observed at two levels of load, i.e., at lower loads, to which textile materials are exposed during processing as well as in further use, and at higher loads. The mechanical properties of textile materials at lower loads can thus be used for process control and optimization, as well as for the development, construction and computer clothing design and the simulation of virtual conventional and intelligent clothing. Namely, the model takes into account the parameters of mechanical properties of textile materials as a complex textile structure, which allow the simulation of falling or drape [1]. Drape as a complex deformation of fabric can be generally defined from two viewpoints, as a two-dimensional or three-dimensional drape. The two-dimensional drape is associated with the deformation caused by gravity acting on a textile surface. Due to its own weight, the fabric bends in one plane, while the three-dimensional drape allows the fabric to be deformed into folds within more than one plane [2]. C.C. Chu defined ‘Drape’ and ‘Drapeability’ as terms for the property of textile materials which allows a fabric to orient itself into graceful folds or pleats when acted upon by the force gravity [3]. In practical terms, it means that the fabric has good drape qualities if the configuration is pleasing to the eye. From this point of view, the word drape is also a qualitative term [2]. Drape parameters can be used to predict the ability to shape and appearance of a garment on the human body and are usually tested using the Cusick drape meter, as a standard and well-known measurement equipment [1,4,5].

Given the increasing application of computer technologies in 2D/3D clothing design, more simple systems for determining the values of mechanical parameters of the fabric have recently been developed, whose parameters can be implemented in CAD systems for 3D prototype development and virtual testing of conventional and intelligent clothing [6,7,8]. However, the Kawabata Evaluation System (KES) is still the best known and most significant system for the objective evaluation of textiles. The main mechanical properties related to the fabric behavior during production and wearing are tensile, bending, shear, and compression properties [1]. Many authors have investigated the influence of the mechanical properties of fabrics on drape [9,10,11,12,13,14]. Geršak, who studied the influence of the elastic potential of fabrics which expresses fabric ability to recover, found that it directly influences the drapeability of the fabric. The results obtained from the relationship between the fabric elastic potential and its fit, i.e., drapeability, indicate that elastic potential influences the drape coefficient and the crease depth. Tensile elastic potential and bending elastic potential directly impact drape, while the influence of shear elastic potential is reflected indirectly through the value of shear hysteresis, known as *2HG5* [15]. Jedda and associates analyzed the relationship between the drape coefficients and the mechanical properties determined by the FAST system and found that the bending and shear stiffness correlate with the drape coefficient better than the thickness [16]. Various prediction models have also been developed which, in addition to the drape coefficient, includes other parameters, such as the number, shape, and dimensions of the folds [13,17,18]. In his research, Jeong found a large variability in the number of folds in the same fabric, which he explains with the influence of the drape speed and the ratio of the sample size to the test disk [19,20]. The development of 3D clothing simulation methods began in the 1990s, with 2D cutting parts simulating their joining around a virtual body model, and surface deformability based on the values of the mechanical properties of the fabric being simulated [21]. The application of virtual 3D technology in the development of new clothing models contributes to greater precision and greatly reduces the cost of making trial models, given the virtual fit testing [22,23,24]. The precisely determined values of the parameters of textile material mechanical properties are a very important factor, i.e., the mathematical model on which the algorithm defining the behavior and deformability of the target material in the simulation is based [22,25], as well as the precise construction of 2D cutting parts and anthropometric characteristics of the virtual body model [24,26].

**Figure 1:**
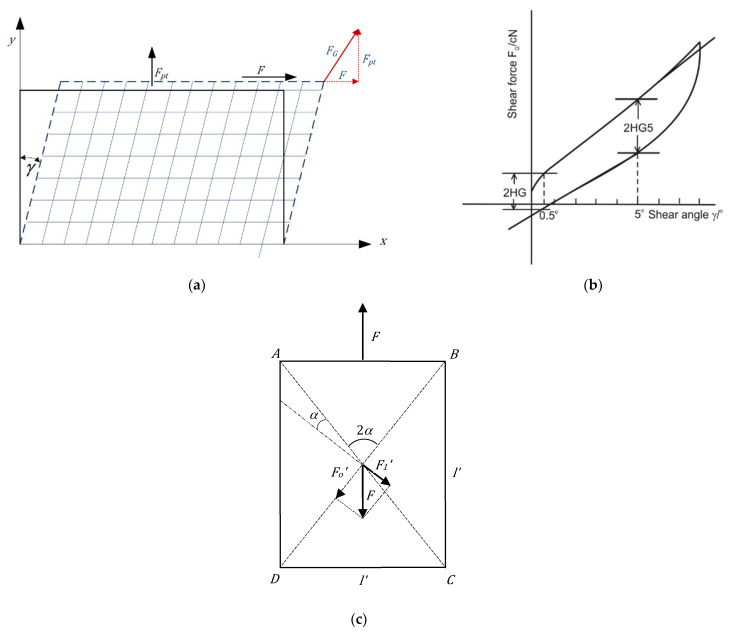
Principle of shear properties measurement: (**a**,**b**) Principles and diagram for KES and (**c**) FAST—Bias extension test.

Clothing simulation models are mostly based on the particle mesh method and the finite element method [23,27]. Considering the complex anisotropic behavior of the textile material, the quadratic particles model and triangular springs mesh allow a better representation of the virtual fabric using objectively determined values of the mechanical properties of the target fabric. Most commercial clothing design and 3D simulation systems allow the entry of the data of mechanical properties determined by one of the two leading objective evaluation systems, KES and/or FAST [25,28,29,30,31]. The relevant parameters of physical and mechanical properties used for simulations are tensile elongations in warp and weft directions, bending rigidity in warp and weft direction, shear rigidity, thickness, and weight. If we compare the KES and FAST system, the KES system allows the determination of the partially and completely reversible deformations caused by small tensile, compression, shear, and bending stresses, while the FAST system measures similar low-stress fabric mechanical properties (compression, bending, extension, and shear), but does not measure recovery properties [1,32]. For example, shear properties measured using the KES system is based on the shear deformation of a specimen. As shown in Figure 1a, constant tension force *F_pt_* is applied along the direction orthogonal to shear deformation and perpendicular to tensile force *F*, respectively. Shear rigidity is defined as the average slope of the curve *F_G_* (γ), (Figure 1b) [1]:

(1) $$\;\;G=tg\;\gamma =\frac{\Delta F_{G}}{\Delta \gamma }$$

where shear force *F_G_* is given by:

(2) $$\;\;F_{G}=F-F_{pt}\cdot tg\;\gamma$$

where *F_G_* is the shear force per unit length, *F_pt_* is the constant tensile force (9.807 cN per unit length), and *γ* is the shear angle (*γ* = 8°).

In the case of the FAST system, it is a bias extension test, in which the warp and weft threads are parallel to the diagonals (Figure 1c) [1]. Applying a force *F*, e.g., in the vertical direction in the figure, the square is deformed into a rectangle, with sides *l*′ and *l*′′, and the threads incline at an angle ±*γ* compared with the vertical (Figure 1c). In the FAST measurement system, simplified formulae are used to calculate shear rigidity *G*, where the deformation is measured as the tangent of the shear angle and can be written as [1]:

(3) $$\;G=\frac{F_{G}}{tg\gamma }=\frac{\frac{F}{2}}{2\cdot e}=\frac{F}{4\cdot e}=\frac{4.9\cdot 100}{4\cdot \varepsilon_{B5}}=\frac{123}{\varepsilon_{B5}}$$

Thus, the application of data determined by the KES system for clothing simulation provides a better assessment of the appearance and fit of a virtual garment model [33]. The application of such a methodology makes it possible to assess the suitability of the target fabric for making a garment model with regard to the desired appearance and fit of the model on the virtual body [25,26].

The aim of the presented research is to determine the correlation between the density of the model polygonal mesh during the simulation and the deformation of the surface to which the mechanical and physical properties of the selected fabrics are assigned. The purpose of the research is to determine the optimal simulation parameters depending on the mechanical properties of the target fabric in order to gain insight into the algorithm that determines the mechanical behavior of the polygonal surface simulating the fabric in a CAD system.

## 2. Research Methodology

An objective evaluation of the parameters of physical and mechanical properties was performed on nine fabrics intended for the production of outerwear. The evaluation of the individual fabrics was carried out with two different systems, Fabric Assurance by Simple Testing (FAST) [34] and Kawabata Evaluation System (KES) [1,35], in order to examine the applicability of each system in the process of computer development and obtaining a realistic 3D model prototype. Complex deformations, i.e., drape of selected fabrics was examined using the Cusick drape meter device, and the values of the determined drape parameters of individual fabrics were used as reference values for evaluating the simulation results. In order to investigate the influence of the polygonal mesh density of digital cutting parts, for each fabric, four drape simulations were performed with a polygon side length variation of two, four, six, and eight millimeters. The obtained computerized drape models were used to analyze the shape and dimensions of the inside curve surface defined by the fabric drape. Model processing and surfaces calculations within the drape curves were performed using the CAD program for 3D modeling Rhinoceros [36]. Based on the determined areas, the drape coefficients were calculated for each simulated fabric in all four variations of the polygonal mesh density. Based on the determined values of the drape coefficients of the simulated fabrics, a correlation analysis was performed with the coefficients of real samples determined using the Cusick drape meter. In this way, the influence of mesh density on the result of 3D simulation was investigated, and the optimal polygon size was defined in relation to the physical and mechanical properties of the target fabric, which gives the results closest to the values of real samples.

### 2.1. Test Fabric Samples

Nine different fabrics intended for making conventional and intelligent outerwear were selected for the research. The selected fabrics differ according to the raw material composition, weave structure, thread density, and fabric weight, which is shown in Table 1. The thread density was determined on conditioned samples by counting the threads in the warp and weft direction using a magnifying glass according to ISO 7211/2-1984 [37]. The fabric weight was determined according to ISO 3801 [38], where five conditioned samples measuring 100 cm^2^ were tested for each fabric, and the fabric mass is expressed as the mean value of individual measurements in g/m^2^.

### 2.2. Determination of the Parameters of Mechanical Properties

Measuring devices of the FAST and KES systems were used to determine the values of the parameters the mechanical properties of the selected fabrics, as seen in Table 2.

From the determined parameter sets of the systems, the following parameters were selected for 3D simulation: extension *EM* by maximum values of *F* (*Fm* = 490.35 cN per unit width) in the warp and weft direction (KES-FB system); extension *E100* by load of 98.07 cN per unit width in the warp and weft direction (FAST system); bending rigidity *B* in the warp and weft direction; shear rigidity *G*; thickness *T0* of fabric at maximum pressure 0.49 cN cm^−2^ (KES-FB system); and the surface layer thickness *ST* as the difference in thickness measured at the two loads (*ST* = *T2–T100*), where *T2* is thickness at 1.96 cN cm^−2^ and *T100* is thickness at 98.07 cNcm^−2^ as a compression property parameter (FAST system) [1,2], as seen in Table 2. The determined parameter values were translated into units suitable for input into a CAD clothing simulation system using the fabric converter [39].

### 2.3. Research on Fabric Drape

**Figure 2:**
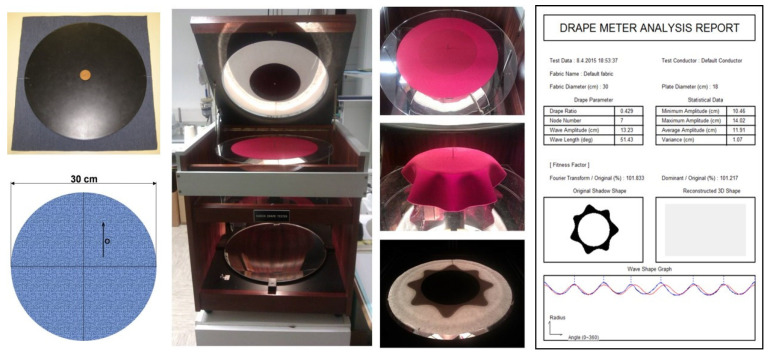
Fabric drape test on a Cusick drape meter.

Fabric drape was investigated using a Cusick drape meter, (Figure 2), on conditioned 30-cm-diameter circular samples, where the measurement for each fabric was performed on five samples, and the results were expressed as the mean of the individual measurements for each fabric [1]. Table 3 presents the captured shaded surfaces of drape fabric samples obtained using Cusick drape meter, based on which the drape coefficients (*K_d_*) were calculated for each fabric according to the Expression (4), where *A* [mm^2^] is the projection area of the deformed fabric shape, *R*_1_ [mm] is the radius of the horizontal disk (90 mm), and *R*_2_ [mm] is the radius of the undeformed sample surface.

(4) $$K_{d}=\frac{A-\pi R_{1}^{2}}{\pi \left(R_{2}^{2}-R_{1}^{2}\right)}$$

### 2.4. Computer Simulation of Fabric Drape

**Figure 3:**
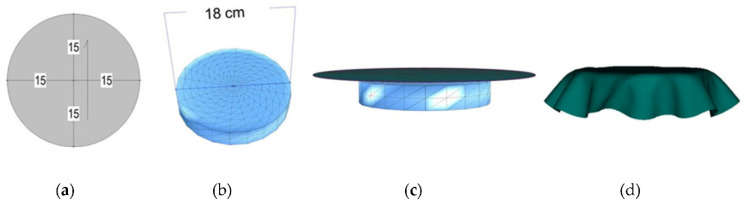
The process of fabric drape computer simulation in the Optitex CAD system: (**a**) creation of a planar circular sample of given dimensions; (**b**) a solid disk model of specified dimensions; (**c**) positioning of the planar circular sample above the disk model; (**d**) computer simulation of fabric drape.

An Optitex CAD system was used for computer simulations of fabric drape [40]. The method of performing simulations and determining the drape coefficients of the simulated samples was defined in accordance with the Cusick drape meter test method in real conditions. For this purpose, circular samples with a diameter of 30 cm were computer constructed (Figure 3a), and the simulations were performed on a solid disk model with a diameter of 18 cm, as found in the Cusick drape meter (Figure 3b). The parameter values of the physical and mechanical properties of the individual fabrics determined with the FAST and KES measuring systems were converted using the fabric editor converter and applied to circular samples for the simulation.

**Figure 4:**
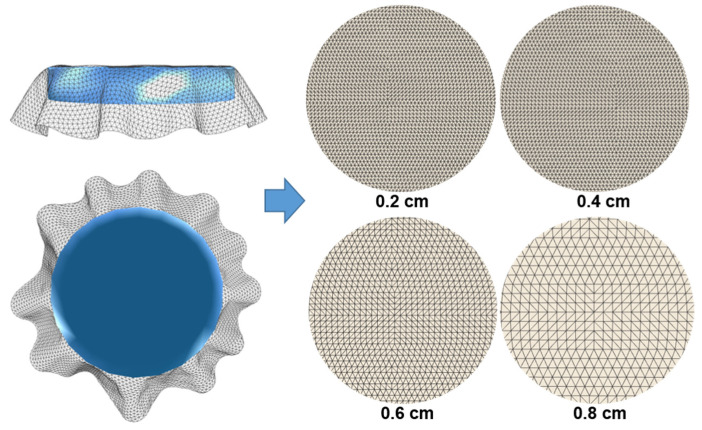
Computer circular fabric samples with different polygonal mesh densities, i.e., polygon side lengths.

During the simulations, the circular samples are positioned exactly in the middle and just above the solid disk (Figure 3c), whereby a free fall of the sample onto the disk is performed and characteristic folds were formed as a shading curve of the fabric drape surface (Figure 3d). To determine the influence of polygonal mesh density on the simulation results, each fabric was simulated four times, with four different polygon dimensions, i.e., with a polygon side length of two, four, six, and eight millimeters, (Figure 4).

**Figure 5:**
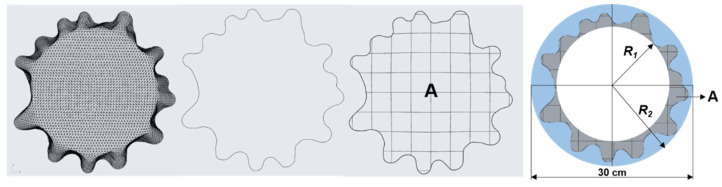
Computer processing of simulated drape samples in the Rhinoceros program.

Computer 3D models of drape samples were stored as surface models suitable for further analysis. The shape and surface analysis of the simulated samples was performed using the Rhinoceros 3D modeling program. In order to determine the drape coefficients on the simulated samples, it is primarily necessary to extract the characteristic drape curve that describes the shape and size of the projected area, and on the basis of which the coefficients are calculated. The edges of the simulated drape models were separated by computer processing, creating 3D curves which describe the edges of the simulated fabric models. The created 3D curves use a 3D flattening method mapped to the *y* plane and closed in the surface (Figure 5), which corresponds and is comparable to the method of testing and determining projected drape surfaces using the Cusick drape meter. Furthermore, computer measurements of surface areas defined by drape curves were performed, and based on the determined values, drape coefficients were calculated for each simulated sample according to the expression (4).

## 3. Results and Discussion

From the results of the drape simulations performed, the influence of the model polygon mesh density on the properties of the simulated surface is visible. By increasing the dimensions of the polygon, i.e., decreasing the density of the polygonal surface, a change in the shape and size of the shaded drape surface can be seen from the presentation of the simulated samples shown in Table 4.

The comparative analysis of the calculated drape coefficients of samples simulated using the values of the mechanical parameters determined by the FAST and KES system and the drape coefficient of the realistically measured samples showed a tendency towards proportionality between the drape parameters and the density parameters of the simulated polygonal surface in both cases. The samples simulated based on the mechanical parameters determined by the KES system showed a significantly higher sensitivity depending on the defined density of the model polygonal mesh compared to samples simulated with the parameters determined by the FAST system.

**Figure 6:**
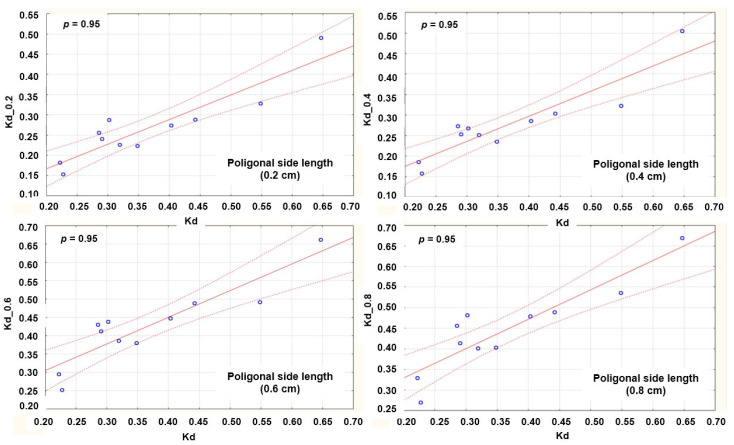
Linear correlation scatter graphs of real and simulated drape coefficients values with different polygonal mesh densities. *p*—reliability, Kd—drape coefficients of real measured fabrics, Kd_0.2—drape coefficients of fabrics simulated with polygonal side length of 2 mm, Kd_0.4—drape coefficients of fabrics simulated with polygonal side length of 4 mm, Kd_0.6—drape coefficients of fabrics simulated with polygonal side length of 6 mm, and Kd_0.8—drape coefficients of fabric simulated with polygonal side length of 8 mm.

Figure 6 presents a scatter graph with 95 percent reliability and shows positive a linear correlation between the real (Kd) and simulated values of drape coefficients (Kd_0.2–Kd_0.8) with different side lengths. A high correlation was found between the values of the drape coefficient of the real and all simulated samples, with slight differences in values depending on the change in the parameters of the polygonal mesh. However, simulations performed using data determined by the KES system show higher values of the determination coefficient (*r*^2^ > 0.8), which is expected, Table 5. Given the visible deviation of the individual samples on the graph (Figure 6), the relationships between the individual values of the drape coefficients and the mechanical properties of an individual fabric sample were further analyzed.

It is evident from the determined values of the drape coefficients of the samples simulated on the basis of mechanical parameters determined by the KES system with different polygonal mesh densities that the surface with higher density and thus higher mobility (polygon side size 0.4 mm) better corresponds to the samples with lower drape coefficient (*Kd* = 0.222–0.319), i.e., soft fabrics that have a nice drop. The lower density of the polygonal mesh of the simulated surface (polygon side size 0.6 mm) corresponds to samples with a higher drape coefficient (*Kd* = 0.348–0.442), i.e., stiffer fabrics that do not have such a nice drop, while for fabrics with a drape coefficient above 0.5 a polygon mesh with a polygon side size of 0.8 mm is most suitable, as seen in Table 6.

Fabric samples F2 and F9 proved to be an exception to the observed behavior during the simulation. In the case of F2 fabric, the best simulation results were with a length of the polygon side of 0.6 cm instead of 0.4 cm, given the measured lower drape coefficient of the real fabric (*Kd* = 0.227). By looking at the values of the parameters of the mechanical properties of the fabric sample F2, one can notice a large value of fabric elongation at the maximum force in the weft direction (*EMT-2* = 31.28%) and very small values of bending rigidity (*B-1* = 0.0085 cNcm, *B-2* = 0.0033 cNcm). The values obtained show that it is a very soft fabric with a large transverse stretch, and, from the aspect of performing numerical simulations, we can say that the fabric is extremely deformable. In this sense, the defined high density of the polygonal model mesh additionally contributes to the mobility of the surface and, as such, is not suitable for such samples because excessive deformations occur. The size of 0.6 cm was determined as the optimal value of the polygon size for the fabric sample F2, as presented in Table 6.

In addition, by considering the values of the parameters of the mechanical properties of fabric sample F9, a high value of fabric thickness (*T0* = 2.42 mm) and higher values of bending rigidity (*B-1* = 0.3354 cNcm, *B-2* = 0.0989 cNcm) can be noticed. The obtained values show that it is a fabric with greater thickness and stiffness, and from the aspect of performing numerical simulations, we can say that the fabric is less deformable. In this sense, the optimal polygon size value for sample F9 was the polygon size of 0.6 cm, not 0.8, which was expected given the large value of the drape coefficient measured on a real fabric sample F9 (*Kd* = 0.647). The lower density of the polygonal surface of the model additionally contributes to the reduced deformability of the surface and, in the case of simulating fabrics with greater thickness and flexural stiffness, prevents real mobility. In this case, the defined smaller dimension of the polygon compensates for the missing surface mobility from the computer aspect and allows for more realistic simulation results (Table 6).

Since the drape is also influenced by other mechanical parameters in addition to the parameters of the bending properties, a correlation analysis was also carried out between the determined values of the mechanical parameters and the drape coefficients of the real and simulated fabric samples, as seen in Table 7.

In this way, the simulated samples gained an insight into the algorithm that determines the mechanical behavior of the polygonal surface that simulates the fabric in the CAD system, which is protected by the manufacturer of the CAD system and is not available because it is a commercial program

For the values of the drape coefficients measured on real samples, a strong correlation was found with the parameters of bending rigidity in both directions and shear rigidity (*r* > 0.8), a medium strong correlation with the parameters of thickness and weight (*r* > 0.6), and a weak correlation with tensile elongation parameters in both directions. If we compare the established correlations of real samples with the correlations of drape coefficients and mechanical parameters of simulated samples, differences in the significance of individual parameters are visible, which can be related to the established differences in the drape visualization of real and simulated samples and determined values of the drape coefficients. The fabric thickness parameter (*T0*) shows a medium–strong correlation (r = 0.74) for the real samples and a strong correlation (*r* = 0.91) for the simulated samples with the drape coefficient, which leads to increased surface stiffness when simulating thicker fabrics, as in the case of the F9 fabric sample. Since the deformability of the surface in the simulation can be directly affected by changing the density parameter of the polygonal mesh, i.e., the length of the polygon side, a smaller optimal polygon size was determined for the F9 fabric sample in relation to the value proportional to the real drape coefficient. This reduced the surface stiffness caused by the excessive influence of the fabric thickness parameter.

In addition, although a weak correlation between drape and tensile parameters was found in both real and simulated samples, higher values of the correlation coefficient can be observed in the simulated samples. The increased influence of tensile parameters on the mechanical behavior of the polygonal surface in the simulation causes excessive mobility, and this may explain the formed shape differences between simulated fabric drape patterns and realistically determined values obtained using Cusick drape meter. An excessive influence of the parameters of tensile properties is especially visible in sample F2, which realistically has a significantly higher value of stretching in the weft direction than the other samples, and this higher value of stretching is further emphasized in relation to the model of mechanical behavior in the simulation, resulting in excessive deformability of the sample.

## 4. Conclusions

Simulations based on the physical and mechanical properties of fabrics allow prediction assessment of the appearance and behavior of products in the development process, thus saving time and costs required for the production of test samples, which is especially important in the development of new models of conventional and intelligent garments. The mesh density of the polygonal model is a very important parameter to obtain the most relevant simulation results.

The investigation revealed that the optimal polygon size for fabrics with a drape coefficient of 0.20 to 0.35 is 0.4 mm, for fabrics with a drape coefficient of 0.35 to 0.50 is 0.6 mm, and for fabrics with a drape coefficient above 0.5 is 0.8 mm. However, it is also necessary to further analyze the individual parameters since significantly increased values of individual parameters can further affect the outcome of the simulation, as is the case with fabrics F2 and F9 from this study.

The research conducted using the CAD system Optitex determined the optimal parameters of simulations depending on the mechanical properties of the target fabric, which facilitates the process of computer prototype development and increases accuracy in analyzing and predicting the appearance and behavior of clothing before making a realistic test model. The use of 3D in the textile industry, especially in the fashion industry, is still too low due to a lack of understanding of the computer simulation evaluation process.

The simulated samples showed good topology results, meaning that the shaded areas are similar in size as the ones measured on real fabric samples, but there are visible shape deviations, where the number of folds differs from the number of folds determined by the Cusick drape meter. The correlation analysis of the projection area surface and the drape coefficient of real and simulated samples revealed a very strong linear correlation, which confirmed the influence of physical and mechanical properties on the fabric simulation, and the obtained results and differences can be used in further research to improve the system and simulation process for more realistic folding. In this sense, given the complexity of fabric drape, which is influenced by numerous parameters, future research will focus on the possibility of analyzing the effects of additional simulation parameters reflected in drape properties.

## Figures and Tables

**Table 1:** Structure of selected fabrics according to raw material composition, wave, thread density, and fabric weight.

Fabric	Raw Material Composition	Wave		Density [Thread/cm]		Weight [g/m^2^]
				Warp	Weft	
F1	100% polyester fiber	Twill 3/1	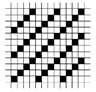	112.2	41.2	135.2
F2	100% polyester fiber	Plane 1/1	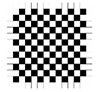	72.8	36.3	83.7
F3	63% polyester fiber, 23% viscose fiber, 4% elastane fiber	Twill 2/1	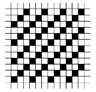	35.7	28.2	286.1
F4	100% wool	Plane 1/1	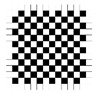	38.9	35.9	161.8
F5	75% wool, 15% polyester fiber, 10% elastane fiber	Twill 2/1	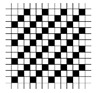	23.7	24.1	178.7
F6	100% polyester fiber	Plane 1/1	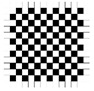	19.6	18.0	159.8
F7	97% polyester fiber, 3% elastane fiber	Plane 1/1	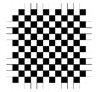	23.2	20.8	178.1
F8	65% cotton, 31% polyester fiber, 4% elastane fiber	Twill 2/1	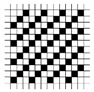	42.6	18.1	253.4
F9	65% polyester fiber, 35% viscose fiber	Plane 1/1	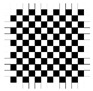	42.7	31.1	366.8

**Table 2:** Values for the mechanical properties of fabrics determined by KES and FAST system (suffix 1 refers to warp direction and suffix 2 to weft direction).

**Measurement system**	**Samples**	**EM-1 [%]**	**EM-2 [%]**	**B-1 [cNcm]**	**B-2 [cNcm]**	**G [cN/(cm°)^−1^]**	**T0 [mm]**
KES	F1	2.75	6.92	0.0195	0.0061	0.41	0.405
	F2	3.90	31.28	0.0085	0.0033	0.46	0.356
	F3	21.54	22.05	0.0297	0.0240	0.61	0.900
	F4	3.02	9.58	0.0324	0.0161	0.73	0.439
	F5	9.05	11.49	0.0214	0.0200	0.50	0.539
	F6	12.41	9.63	0.0362	0.3980	0.49	0.600
	F7	7.02	7.75	0.0532	0.4440	0.69	0.510
	F8	3.48	21.15	0.0743	0.0370	1.26	0.980
	F9	3.97	6.27	0.3354	0.0989	1.15	2.420
**Measurement system**	**Samples**	**E100-1 [%]**	**E100-2 [%]**	**B-1 [µNm]**	**B-2 [µNm]**	**G [Nm^−1^]**	**ST [mm]**
FAST	F1	1.1	3.9	4.5	1.9	23	0.060
	F2	1.6	18.0	2.1	1.1	16	0.080
	F3	11.9	11.7	7.7	6.3	27	0.174
	F4	1.4	4.9	7.6	3.9	33	0.082
	F5	4.9	5.9	5.7	5.3	24	0.086
	F6	6.5	5.1	9.1	9.3	24	0.096
	F7	3.2	3.4	11.9	9.8	38	0.072
	F8	1.6	10.6	32.1	10.3	60	0.225
	F9	1.5	2.8	94.3	23.4	67	0.960

**Table 3:** Measured values of drape coefficient for nine selected fabrics (F1–F9).

Samples	F1	F2	F3	F4	F5	F6	F7	F8	F9
	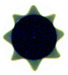	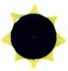	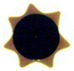	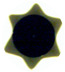	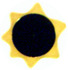	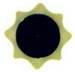	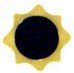	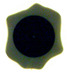	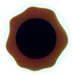
Kd	0.222	0.227	0.285	0.319	0.348	0.403	0.442	0.548	0.647

**Table 4:** Computer simulations of drape fabric samples with different polygonal mesh densities.

Samples	FAST				KES			
	0.2 mm	0.4 mm	0.6 mm	0.8 mm	0.2 mm	0.4 mm	0.6 mm	0.8 mm
F1	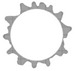	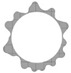	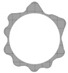	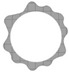	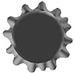	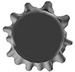	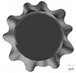	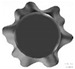
F2	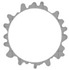	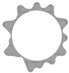	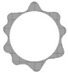	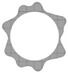	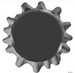	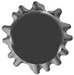	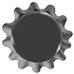	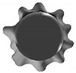
F3	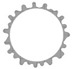	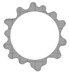	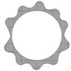	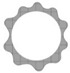	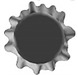	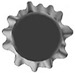	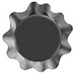	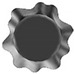
F4	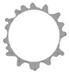	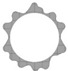	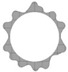	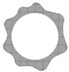	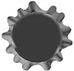	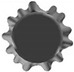	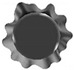	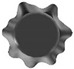
F5	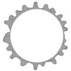	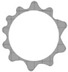	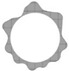	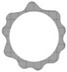	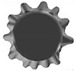	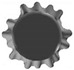	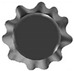	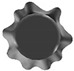
F6	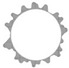	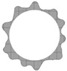	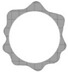	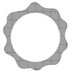	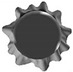	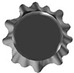	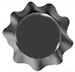	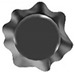
F7	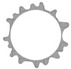	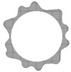	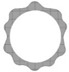	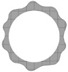	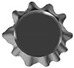	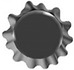	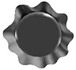	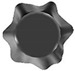
F8	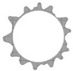	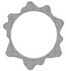	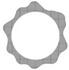	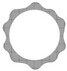	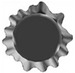	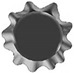	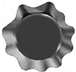	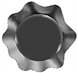
F9	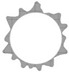	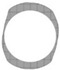	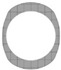	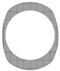	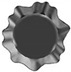	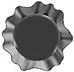	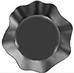	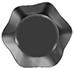

**Table 5:** Linear correlation analysis between the values of the drape coefficients of real fabrics and fabrics simulated with different polygonal mesh densities.

Measurement System	FAST				KES			
Correlation parameters	K_d_ 0.2	K_d_ 0.4	K_d_ 0.6	K_d_ 0.8	K_d_ 0.2	K_d_ 0.4	K_d_ 0.6	K_d_ 0.8
*r*	0.868	0.861	0.897	0.915	0.910	0.912	0.900	0.899
*r* ^2^	0.754	0.742	0.805	0.839	0.828	0.831	0.810	0.809
*t*	5.250	5.090	6.093	6.841	6.591	6.649	6.197	6.186
*p*	0.001	0.001	0.000	0.000	0.000	0.000	0.000	0.000
*a*	0.037	0.026	0.272	0.305	0.046	0.052	0.159	0.187
*b*	0.234	0.946	0.750	0.747	0.607	0.612	0.726	0.712

*r*—Pearson correlation coefficient, *r*^2^—determination coefficient, *t*—value to test the significance of the correlation coefficient, *p*—level of correlation significance, *a*—regression constant (value of the dependent variable when the value of the independent variable is equal to 0), and *b*—regression coefficient (average change in the dependent variable value for the unit change in the independent variable).

**Table 6:** Values of drape coefficients of simulated samples.

Samples	Drape	Simulation FAST Data				Simulation KES Data			
	K_d_	K_d_ 0.2	K_d_ 0.4	K_d_ 0.6	K_d_ 0.8	K_d_ 0.2	K_d_ 0.4	K_d_ 0.6	K_d_ 0.8
F1	0.222	0.113	0.319	0.492	0.530	0.182	0.186	0.295	0.329
F2	0.227	0.107	0.260	0.453	0.465	0.153	0.158	0.252	0.270
F3	0.285	0.080	0.258	0.434	0.466	0.256	0.273	0.431	0.457
F4	0.319	0.125	0.354	0.528	0.565	0.226	0.252	0.386	0.402
F5	0.348	0.108	0.315	0.507	0.556	0.224	0.235	0.380	0.403
F6	0.403	0.149	0.390	0.575	0.623	0.274	0.286	0.448	0.479
F7	0.442	0.141	0.411	0.611	0.642	0.289	0.304	0.489	0.490
F8	0.548	0.149	0.399	0.583	0.628	0.328	0.323	0.492	0.536
F9	0.647	0.203	0.785	0.840	0.853	0.491	0.505	0.662	0.669

**Table 7:** Linear correlation analysis of the parameters of physical and mechanical properties influences the value of the drape coefficient of real and simulated fabrics.

Drape Coefficient	Correlation Parameters	Mechanical Parameters from KES System						
		T0	B-1	B-2	EMT-1	EMT-2	G	W
Kd_real_	*r*	0.740	0.849	0.916	−0.250	−0.411	0.884	0.642
	*p*	0.009	0.001	0.000	0.459	0.209	0.000	0.033
	*a*	0.292	0.292	0.212	0.406	0.458	0.117	0.147
	*b*	0.381	0.004	0.020	−0.010	−0.012	0.007	0.106
Kd_sim0.4_	*r*	0.911	0.955	0.963	−0.352	−0.541	0.775	0.604
	*p*	0.000	0.000	0.000	0.288	0.086	0.005	0.049
	*a*	0.272	0.281	0.199	0.434	0.504	0.132	0.145
	*b*	0.514	0.005	0.023	−0.015	−0.017	0.007	0.110
Kd_sim0.6_	*r*	0.882	0.932	0.928	−0.368	−0.557	0.756	0.552
	*p*	0.000	0.000	0.000	0.266	0.075	0.007	0.078
	*a*	0.399	0.406	0.333	0.541	0.604	0.278	0.302
	*b*	0.438	0.004	0.020	−0.014	−0.015	0.006	0.088

## Data Availability

Data available in a publicly accessible repository.

## References

[1] Geršak J. (2013). Objektivno Vrednovanje Plošnih Tekstilija i Odjeće.

[2] Geršak J. (2013). Study of the complex deformations of textile structure. Complex Fabric Deformations and Clothing Modelling in 3D.

[3] Chu C.C., Cummings C.L., Teixeriara N.A. (1950). Mechanics of Elastic Performance of Textile Material, Part V: A Study of the Factors Affecting the Drape of Fabric, Development of a Drape Meter. Text. Res. J..

[4] Cusick G.E. (1965). The dependence of fabric drape on bending and shear stiffness. J. Text. Inst..

[5] Cusick G.E. (1968). The measurement of fabric drape. J. Text. Inst..

[6] Kujipers S., Luible Bär C., Hugh Gong R. (2020). The measurement of fabric properties for virtual simulation—A critical review. IEEE SA Industry Connections.

[7] Strazdiene E. (2011). Textiles Objective and Sensory Evaluation in Rapid Prototyping. Mater. Sci..

[8] Pavlinic D.Z., Gersak J. (2004). Design of the System for Prediction of Fabric Behavior in Garment Manufacturing Processes. Int. J. Cloth. Sci. Technol..

[9] Peirce F.T. (1930). The Handle of Cloth as a Measurable Quantity. J. Text. Inst..

[10] Abbott N.J. (1951). The Measurement of Stiffness in Textile Fabrics. Text. Res. J..

[11] Niwa M., Seto F. (1986). Relationship between Drapeability and Mechanical Properties of Fabrics. J. Text. Mach. Soc. Jpn..

[12] Jevšnik S., Geršak J., Gubenšek I. (2005). The advance engineering methods to plan the behavior of fused panel. Int. J. Cloth. Sci. Technol..

[13] Robson D., Long C.C. (2000). Drape Analysis using Imaging Techniques. Cloth. Text. Res. J..

[14] Kenkare N., Plumlee T.M. (2005). Fabric drape measurement: A modified method using digital image processing. J. Text. Appar. Technol. Manag..

[15] Geršak J. (2004). Study of relationship between fabric elastic potential and garment appearance quality. Int. J. Cloth. Sci. Technol..

[16] Jedda H., Ghith A., Sakli F. (2007). Prediction of fabric drape using the FAST system. J. Text. Inst..

[17] Chu C.C. (2017). Determination of Factors Which Influence the Draping Properties of Cotton Fabrics.

[18] Lo W.M., Hu J.L., Li L.K. (2002). Modeling a Fabric Profile. Text. Res. J..

[19] Jeong Y.J. (1998). A Study of Fabric-Drape Behavior with Image Analysis Part I: Measurement, Characterization, and Instability. J. Text. Inst..

[20] Jeong Y.J., Philips D.G. (1998). A study of Fabric Drape Behavior with Image Analysis, Part II: The Effect of Fabric Structure and Mechanical Properties on Fabric Drape. J. Text. Inst..

[21] Sayem A.S.M., Kennon R., Clarke N. (2010). 3D CAD systems for the clothing industry. Int. J. Fash. Des. Technol. Educ..

[22] Luible C. (2008). Study of Mechanical Properties in the Simulation of 3D Garments. Ph.D. Thesis.

[23] Volino P., Magnenat-Thalmann N. (2000). Virtual Clothing: Theory and Practice.

[24] Kuijpers A.A.M., Gong R.H. (2014). Virtual tailoring for enhancing product development and sales. Proceedings of the 4th International Global Fashion Conference: Re-Thinking and Reworking Fashion.

[25] Ancutiene K., Strazdiene E., Lekeckas K. (2014). Quality evaluation of the appearance of virtual close-fitting woven garments. J. Text. Inst..

[26] Petrak S., Mahnic M., Rogale D. (2015). Impact of male body posture and shape on design and garment fit. Fibres Text. East. Eur..

[27] Rizzi C., Fontana M., Cugini U. (2004). Towards virtual prototyping of complex-shaped multi-layered apparel. Comput. Aided. Des. Appl..

[28] Pandurangan P., Eischen J., Kenkare N., Lamar T.A.M. (2008). Enhancing accuracy of drape simulation. Part II: Optimized drape simulation using industry-specific software. J. Text. Inst..

[29] Lim H.S. (2009). Three Dimensional Virtual Try-on Technologies in the Achievement and Testing of Fit for Mass Customization. Ph.D. Thesis.

[30] Wu Y.Y., Mok P.Y., Kwok Y.l., Fan J.T., Xin J.H. (2011). An investigation on the validity of 3D clothing simulation for garment fit evaluation. Proceedings of the IMProVe 2011 International Conference on Innovative Methods in Product Design.

[31] Power J. (2013). Fabric objective measurements for commercial 3D virtual garment simulation. Int. J. Cloth. Sci. Technol..

[32] Luible C., Magnenat-Thalmann N. Suitability of Standard Fabric Characterisation Experiments for the Use in Virtual Simulations. http://citeseerx.ist.psu.edu/viewdoc/download?doi=10.1.1.524.2550&rep=rep1&type=pdf.

[33] Kenkare N., Lamar T.A.M., Pandurangan P., Eischen J. (2008). Enhancing accuracy of drape simulation, Part 1: Investigation of drape variability via 3D scanning. J. Text. Inst..

[34] De Boos A. (1994). Tester, D. SiroFAST Fabric Assurance by Simple Testing. A System for Fabric Objective Measurement and Its Application in Fabric and Garment Manufacture.

[35] Kawabata S. (1980). The Standardisation and Analysis of Hand Evaluation.

[36] Becker M., Golay P. (1999). Rhino–Nurbs 3D Modeling.

[37] (2017). ISO 7211/2-1984 Textiles–Woven Fabrics–Construction–Methods of Analysis, Part 2: Determination of Number of Threads per Unit Length. 1984. https://www.iso.org/standard/13842.html.

[38] (2017). ISO 3801:1977 Textiles–Woven Fabrics–Determination of Mass per Unit Length and Mass per Unit Area. 1977. https://www.iso.org/standard/9335.html.

[39] Fabric Converter (2012). Optitex Technical Documentation.

[40] Optitex. https://optitex.com/.

